# Population tobacco control interventions and their effects on social inequalities in smoking: placing an equity lens on existing systematic reviews

**DOI:** 10.1186/1471-2458-8-178

**Published:** 2008-05-27

**Authors:** Caroline Main, Sian Thomas, David Ogilvie, Lisa Stirk, Mark Petticrew, Margaret Whitehead, Amanda Sowden

**Affiliations:** 1 Peninsula Technology Assessment Group, Peninsula College of Medicine and Dentistry, University of Exeter, Exeter, UK; 2 Medical Research Council Social and Public Health Sciences Unit, Glasgow, UK; 3 Medical Research Council Epidemiology Unit, Cambridge, UK; 4 Centre for Reviews and Dissemination, University of York, York, UK; 5 Public and Environmental Health Research Unit, London School of Hygiene and Tropical Medicine, London, UK; 6 Department of Public Health, University of Liverpool, Liverpool, UK

## Abstract

**Background:**

With smoking increasingly confined to lower socio-economic groups, the tobacco control community has been urged to identify which population-level tobacco control interventions work in order to help tackle smoking-related health inequalities. Systematic reviews have a crucial role to play in this task. This overview was therefore carried out in order to (i) summarise the evidence from existing systematic reviews of population-level tobacco control interventions, and (ii) assess the need for a new systematic review of primary studies, with the aim of assessing the differential effects of such interventions.

**Methods:**

Systematic review methods were used to evaluate existing systematic reviews that assessed a population-level tobacco control intervention and which reported characteristics of included participants in terms of at least one socio-demographic or socio-economic factor.

**Results:**

Nineteen systematic reviews were included. Four reviews assessed interventions aimed at the population level alone, whilst fifteen included at least one primary study that examined this type of intervention. Four reviews assessed youth access restrictions, one assessed the effects of increasing the unit price of tobacco, and six assessed smoking bans or restrictions. Of the eight remaining reviews, six assessed multi-component community based interventions, in which the population-level interventions were part of a wider tobacco control programme, and two assessed the impact of smoking bans or restrictions in reducing exposure to environmental tobacco smoke. We found tentative evidence that the effect of increasing the unit price of tobacco products may vary between ethnic and socio-economic groups, and between males and females. However, differences in the context and the results of different reviews made it difficult to draw any firm conclusions. Few identified reviews explicitly attempted to examine differences in intervention effects between socio-demographic groups. Therefore on the basis of these reviews the potential for smoking bans, and youth access restrictions to decrease social inequalities in smoking remains unknown.

**Conclusion:**

There is preliminary evidence that increases in the unit price of tobacco may have the potential to reduce smoking related health inequalities. There is a need for equity effects to be explicitly evaluated in future systematic reviews and in primary research assessing the effects of population tobacco control interventions.

## Background

In countries with a mature smoking epidemic, smoking is persistently associated with lower socio-economic status (SES), where SES is defined by one or more indices of social deprivation. In 2004, for example, within the United Kingdom (UK), 32% of men and 30% of women in routine and manual occupations smoked compared with 20% and 17% of their respective professional counterparts [1]. However, although these figures highlight the current social gradient in smoking prevalence, they also disguise the very high prevalence rates amongst the most socio-economically deprived groups, among whom prevalence rates can reach over 70 per cent [2].

Reducing social inequalities in smoking and its health consequences is now a public health [3] and political priority [4], and the Department of Health in the UK has set a specific target to reduce the prevalence of smoking in "manual groups" from 32% to 26% by 2015 [5]. With smoking increasingly confined to lower socio-economic groups, the tobacco control community is therefore being urged to identify "what messages and interventions work to get lower socio-economic groups to stop smoking" [6].

While we already have a wealth of evidence about the effects of measures to reduce smoking, many of these involve services targeted at the individual – an approach to promoting health which some sections of the population are more likely to take up or successfully engage with than others [7,8]. For example, a recent evaluation of National Health Service (NHS) smoking cessation services has shown that although services successfully reached smokers in the lowest socio-economic group, the quit rate for these smokers was only half that achieved in the highest socio-economic group [9,10]. As the demographic profile of current smokers changes due to lower entry rates into and higher cessation rates from smoking among more socio-economically advantaged groups, a more disadvantaged and potentially more nicotine-dependent smoking population may be left behind. It can therefore be anticipated that successful interventions for reducing smoking rates in the past may fail to achieve the same results with current and future generations of smokers. It may therefore be particularly important to address the macro-level or "upstream" determinants of smoking concomitantly with proximal determinants, since intervening at this level may have greater potential to influence larger numbers of people and reduce the "smoking gap".

Evidence about the effects of such interventions should ideally be derived from systematic reviews. However, the lack of relevant systematic reviews to inform the landmark 1998 Acheson report on health inequalities has been noted [11,12]. Our own pilot study [13] and a Health Development Agency (HDA) report [14] did, however, suggest that there may be review-level evidence on inequalities in smoking. In particular the HDA report suggested that there may be value in revisiting the primary studies to determine whether they can provide sufficient data stratified by high risk, vulnerable, low socio-economic, low education, or minority ethnic groups. However this is a challenging task as it requires re-reviewing a very sizeable and heterogeneous literature. As a step towards achieving this goal, we carried out a comprehensive overview of the evidence obtained from existing systematic reviews. In order to reduce the risk of missing potentially relevant review-level evidence we included reviews that were "borderline" systematic, as described in the methods. Our overall aim was to determine what could be inferred from existing reviews about the effects of tobacco control interventions on social inequalities in smoking, and to provide the groundwork for any subsequent systematic reviews.

## Methods

### Search methods

We searched electronic databases and library catalogues, bibliographies and reference lists for published and unpublished systematic reviews and "borderline systematic" reviews of the effects of any type of intervention to prevent or reduce smoking, access to tobacco products, or exposure to environmental tobacco smoke. The search syntax included groups of terms for "smoking" and "review". No language restrictions were applied. Further details of the search strategy are available from the authors.

### Inclusion criteria

We included reviews of the effects of population-level tobacco control interventions which reported characteristics of the participants in at least some of the included primary studies in terms of at least one socio-demographic variable (sex, race or ethnicity), socio-economic status (occupation, educational level or income), religion, place of residence or area-level index of deprivation. Age was also included as a socio-demographic factor if the intervention targeted vulnerable age groups (adolescents or young adults).

We defined population-level tobacco control interventions as those applied to populations, groups, areas, jurisdictions or institutions with the aim of changing the social, physical, economic or legislative environment to make them less conducive to smoking. These are approaches that mainly rely on state or institutional control, either of a link in the supply chain or of smokers' behaviour in the presence of others. Examples include tobacco crop substitution or diversification, removing subsidies on tobacco production, restricting trade in tobacco products, measures to prevent smuggling, measures to reduce illicit cross-border shopping, restricting advertising of tobacco products, (enforcing) restrictions on selling tobacco products to minors, mandatory health warning labels on tobacco products, increasing the price of tobacco products, restricting access to cigarette vending machines, restricting smoking in the workplace, and restricting smoking in public places. Such approaches could also form part of wider, multifaceted interventions in schools, workplaces or communities. We did not include interventions whose main aim was to strengthen the capacity of individuals to stop smoking or to resist taking up smoking, even if these interventions were applied to whole groups or populations (e.g. mass media health education campaigns). These are approaches that mainly rely on individuals engaging voluntarily with measures intended to help them.

To be eligible for inclusion in our analysis, reviews had to meet the two mandatory criteria for admission to the Database of Abstracts of Reviews of Effects (DARE) [15]: they had to address a clearly defined question, and to have made an effort to identify all relevant literature by searching at least one named database combined with either checking references, hand-searching journals, citation searching, or contacting authors in the field. We defined reviews as "systematic" if at least two components (interventions, participants, outcomes, or study designs) of the review question were explicitly defined and the search criteria were met, and as "borderline systematic" if two or more components of the review question could be inferred from the title or text.

### Evaluation of included reviews

**Figure 1:**
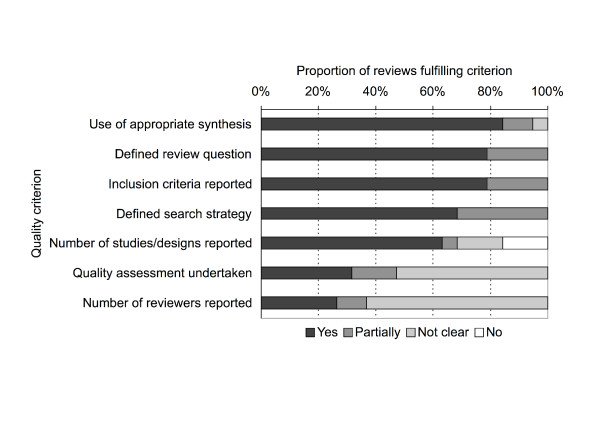
Quality assessment of included reviews.

Two reviewers independently screened abstracts for inclusion, extracted data, and quality assessed all included reviews using a checklist adapted from the seven criteria used for DARE [15] (Figure 1). We classified reviews into five categories of intervention: youth access restrictions, increasing the unit price of tobacco products, multi-component community-based programmes, smoking bans and restrictions, and interventions to prevent exposure to environmental tobacco smoke. Where reviews covered more than one type of intervention, the dominant area determined its classification in our summary. An overview of the key characteristics of each review is available as an additional file 1. We then grouped reviews according to the intervention type, and the participants and combined the results in a narrative synthesis.

## Results

### Quantity and quality of evidence

We found 176 systematic and "borderline systematic" reviews of the effects of interventions on smoking, access to tobacco products or exposure to environmental tobacco smoke. Of these, only 25 (14%) addressed the effects of population-level tobacco control interventions [16-40]. Nineteen of these reviews, ranging in quality (Figure 1) from four "borderline" reviews to four high-quality Cochrane reviews, reported socio-demographic data of some kind and were included in our analysis [16-34]. These reviews included the results of 581 primary studies in total of which 82 had been included in more than one review. No study was included in more than five reviews, with the majority of the 82 studies included in two reviews. Some reviews were focused on a specific type of intervention, others had a broader focus such as community-based interventions or reducing exposure to environmental tobacco smoke. Only three of the 19 reviews explicitly aimed to assess if effects of interventions varied between socio-demographic groups [29,32,33]. The others focused on specific at-risk socio-demographic groups or reported that some of their included primary studies had such a focus.

### Findings of the reviews

Across the 19 included reviews, we found evidence about the effects of three types of population-level tobacco control intervention.

### Increasing the price of tobacco products

We found two reviews, both dealing specifically with young people, one based exclusively on US data [20] and one from the UK [28]. The US review found evidence that higher prices for tobacco products were associated with lower overall levels of smoking uptake and tobacco consumption by both adolescents and young adults. Four primary studies in this review included stratified analyses showing differential effects by ethnic group or sex. Two of these studies provided tentative evidence that young black Americans were more responsive to price than their white counterparts [41,42]. The review also concluded that males were more responsive to price than females [41-44]. In contrast, the UK data showed that females in all socio-economic groups were more responsive to price than their male counterparts [45]. In the lowest socio-economic group, however, smoking prevalence in both males and females was significantly associated with price.

### Restricting young people's access to tobacco products

We found six reviews dealing with education, law enforcement, community mobilisation, or combinations of these approaches to deter retailers from selling tobacco to minors or allowing them access to vending machines [16-19,28,30]. These reviews found that enforced controls on retailers could reduce illegal under-age sales, but evidence of any effect on actual smoking behaviour was equivocal both within and between reviews [16-19,28,30]. The majority of voluntary agreements with retailers had no effect even on sales. Although five reviews reported differences in the age, sex or ethnicity of participants between studies [16,18,19,28,30], none reported whether the effects of interventions varied according to these individual characteristics; nor could we deduce from these reviews whether the effects of access controls varied according to area-level socio-economic characteristics.

### Restricting or banning smoking

We found 11 reviews examining the effects of smoking bans or restrictions in a variety of population groups including adolescents, students in higher education, employees, Indigenous Australians, and people being treated for mental illness or substance misuse [21-25,28-30,33,34]. Bans or restrictions were associated with reduced cigarette consumption at work or school [24-26,28,29], but evidence of a reduction in overall consumption was less clear. Two primary studies indicated more comprehensive policies were associated with lower consumption by students both in and outside school and college [28]. Four reviews examined the effects of bans or restrictions on exposure to environmental tobacco smoke [24,26,33,34] and found significant improvements in nicotine vapour levels, smoke exposure and air quality in both workplaces and public places [24,26,34]. Two reviews aimed to produce stratified estimates of effects [29,33]. One included a primary study with results stratified by sex [29]. This provided tentative evidence that girls were more responsive to school-wide smoking policies than boys [29]. The second review failed to find any differential effects for the population-level tobacco control interventions [33]. Although a further six reviews reported differences in the age, sex, ethnicity or occupational status of participants between studies [22-25,28,30], none reported whether the effects of interventions varied according to these individual characteristics.

## Discussion

Health professionals, researchers and policymakers alike need to know how social inequalities in smoking can be reduced [3,6]. It is often stated that systematic reviews evaluating the effects of interventions might be able to help answer this question. In practice however, we discovered that when the findings of existing reviews are viewed through "an equity lens" – that is, when we attempted to use them to answer a new research question about reducing inequalities – there were relatively few data. Nonetheless, there are both positive messages about the effects of interventions to be gleaned, as well as pointers towards future systematic reviews in the field of tobacco control.

Firstly, most systematic reviews in this field have focused on "downstream" measures aimed at changing individual smoking behaviour – an illustration of an "inverse evidence" law whereby we know least about the effects of interventions most likely to influence the health of the largest number of people [46,47]. Of the few reviews that have examined the effects of "upstream" tobacco control measures, only three set out to consider how those effects vary between socio-demographic groups [29,32,33]. These reviews considered either sex [29,32], or sex and age [33]. Ironically, the best available evidence about differential effects came from two reviews that had not explicitly set out to address this question [20,28]. These reviews offered tentative evidence that the effect of increasing the unit price of tobacco varies between ethnic [41,42] and socio-economic groups [45] and between the sexes [41-44]. We also found preliminary evidence suggesting that the effects of school-wide smoking policies may vary between the sexes [29]. However, it was not possible to assess how these findings might apply to schools where more stringent tobacco control measures have already been implemented.

We chose to focus on what could be gleaned from existing systematic reviews about the effects of population-level tobacco control interventions on social inequalities in smoking. From our overview, the evidence suggests that a variety of interventions may be effective in influencing a range of smoking related outcomes, but that these effects are generally presented as averages across the entire population, and existing systematic reviews tend not to indicate whether the effects vary for different sub-groups. In future, systematic reviewers should where possible extract data on the differential effects of interventions as well as on the population-level effects. Crucially this depends on such data being available in the primary studies, and it is likely that in many cases these data on distributive effects are not reported, or are not analysed [13].

More positively this examination of existing reviews does indicate that the effects of population-level tobacco control interventions have been studied in populations with different age, sex, ethnic and occupational characteristics. This indicates that there is real potential to uncover new insights from existing data by re-examining the primary studies. We have subsequently undertaken such a systematic review [48].

## Conclusion

There is preliminary evidence that increases in the unit price of tobacco may have the potential to reduce smoking related health inequalities. Equity effects should be explicitly routinely evaluated in both systematic reviews and primary research assessing the effects of population tobacco control interventions.

## Competing interests

The authors declare that they have no competing interests.

## Authors' contributions

DO, AS, MP and MW designed the study. LS conducted the searches. CM and ST screened the search results, assessed studies for inclusion, conducted data extraction and quality assessment and synthesised the data. All authors contributed to the interpretation of findings for research and policy. CM wrote the first draft of the manuscript and all other authors contributed to its critical revision and approved the final version. CM is guarantor.

## Pre-publication history

The pre-publication history for this paper can be accessed here:



## Supplementary Material

Additional file 1 Table of selected characteristics of included reviews. Click here for file
