# Supplementary material for: Population tobacco control interventions and their effects on social inequalities in smoking: placing an equity lens on existing systematic reviews
Source: BMC Public Health. 2008 May 27;8:178. doi: 10.1186/1471-2458-8-178 (PMC2412872; doi:10.1186/1471-2458-8-178)
Supplement: Additional file 1 — Table of selected characteristics of included reviews. [file 1471-2458-8-178-S1.pdf]

Table of selected characteristics of included reviews*

Review	Scope	Age group	Focus	Full systematic review	Explicit consideration of health inequalities	Discussed differential intervention effects
Levy (2002)[16]	Youth access restrictions	Adolescents	Both	—	—	+
Lund (1999)[17]	Youth access restrictions	Adolescents	Smoking cessation	—	—	—
Stead (2002) [18]	Youth access restrictions	Adolescents	Both	+	—	+
Fichtenberg (2002)[19]	Youth access restrictions	Adolescents	Smoking cessation	+	—	+
Hopkins (2001)[20]	Increasing unit price of tobacco	Adolescents	Smoking cessation	+	—	+
Murphy-Hoefer (2005)[21]	Smoking bans or restrictions	Adolescents	Both	+	—	—
El-Guebaly (2002)[22]	Smoking bans or restrictions	Adults	Smoking cessation	+	—	+
Ivers (2003)[23]	Smoking bans or restrictions	Adults	Both	+	—	+
Moher (2003)[b][24]	Smoking bans or restrictions	Adults	Smoking cessation	+	—	+
Fichtenberg (2002)[25]	Smoking bans or restrictions	Adults	Smoking cessation	+	—	+
Eriksen (1998)[b][26]	Smoking bans or restrictions	Adults	Smoking cessation	+	—	—
Sowden (2003)[c][27]	Community-based programmes[a]	Adolescents	Smoking prevention	+	—	+
Stead (1995)[28]	Community-based programmes[a]	Adolescents	Smoking prevention	—	—	+
Blake (2001)[29]	Community-based programmes[a]	Adolescents	Smoking prevention	—	+	+
Wakefield (2000)[30]	Community-based programmes[a]	Adolescents	Both	+	—	+
Friend (2002)[c][31]	Community-based programmes[a]	Adults	Both	+	—	—
Secker-Walker (2002) [c] [32]	Community-based programmes[a]	Adults	Smoking cessation	+	+	+
Roseby (2002)[33]	Reductions in ETS	Adults	Both	+	+	+
Serra (2002)[34]	Reductions in ETS	Adults	Smoking cessation	+	—	—

* Where reviews covered more than one type of intervention the dominant area determined the classification; ETS: environmental tobacco smoke; Both: focused on smoking cessation and prevention; [a] These reviews included one or more primary studies that assessed the effects of increasing the unit price of tobacco, youth access restrictions, or smoking bans and restrictions. The overall scope of these reviews concerned multi-component community-based programmes and they therefore could include primary studies of interventions not classified as population-level tobacco control interventions. Results from the primary studies are discussed in the text under the relevant type of intervention; [b] Some primary studies included in the review reported outcomes for reductions in ETS; [c] Outcomes not reported separately for population tobacco control interventions.
